# Evolved bacterial siderophore-mediated antibiotic cross-protection

**DOI:** 10.21203/rs.3.rs-2644953/v1

**Published:** 2023-05-18

**Authors:** Anna Clara M. Galdino, Mylene Vaillancourt, Diana Celedonio, Kara Huse, Yohei Doi, Janet S. Lee, Peter Jorth

**Affiliations:** 1 Department of Pathology and Laboratory Medicine, Cedars-Sinai Medical Center, Los Angeles, CA, 90048 USA; 2 Acute Lung Injury Center of Excellence, Division of Pulmonary, Allergy, and Critical Care Medicine, Department of Medicine; Vascular Medicine Institute, University of Pittsburgh, Pittsburgh, PA 15213, USA; 3 Department of Medicine, Department of Biomedical Sciences, Cedars-Sinai Medical Center, Los Angeles, CA, 90048 USA

## Abstract

Antibiotic cross-protection enables resistant bacteria to protect other bacteria that would be otherwise susceptible to the drug. Cefiderocol is the first siderophore cephalosporin antibiotic approved for treating Gram-negative bacterial infections, including carbapenem-resistant *Pseudomonas aeruginosa* strains. While highly effective, CFDC resistance has been detected clinically, and mechanisms of resistance and cross-protection are not completely understood. In this study, we used experimental evolution and whole genome sequencing to identify cefiderocol resistance mechanisms and evaluated the trade-offs of evolving resistance. We found some cefiderocol-resistant populations evolved cross-protective social behavior, preventing cefiderocol killing of susceptible siblings. Notably, cross-protection was driven by increased secretion of bacterial iron-binding siderophores, which is unique from previously described antibiotic degradation mediated cross-protection. While concerning, we also showed that resistance can be selected against in drug-free environments. Deciphering the costs associated with antibiotic resistance might aid the development of evolution-informed therapeutic approaches to delay the evolution of antibiotic resistance.

Antibiotic cross-protection occurs when resistant bacteria protect susceptible bacteria from antibiotic killing and is typically driven by antibiotic degradation by protector cells^1,2^. Cefiderocol is a siderophore cephalosporin antibiotic that acts as a trojan-horse, hijacking bacterial iron transporters to facilitate its uptake^3^. While cefiderocol has potent activity against multidrug resistant bacteria^4,5^, mechanisms of resistance and cross-protection are poorly characterized. Using experimental evolution and whole genome sequencing, we identified mutations underlying cefiderocol resistance in *Pseudomonas aeruginosa* in synthetic cystic fibrosis sputum^6^ and human urine media. Resistance arose through multiple unique mutations, including distinct mutations in cystic fibrosis and urine media. While evolved resistance paid fitness costs which caused growth defects in the absence of cefiderocol, some resistant populations evolved benevolent cross-protective behavior rescuing susceptible siblings from killing. Here we show that cefiderocol cross-protection was driven by mutation of a two-component sensor, increasing *P. aeruginosa* siderophore secretion which chelated iron from cefiderocol. Because iron-free cefiderocol is not taken up by bacteria^3^, these data show that antibiotic cross-protection can occur via degradation-independent mechanisms. These findings suggest that antibiotic cycling could be beneficial for cefiderocol clinically: resistant bacteria could protect susceptible siblings, and protected cells could outcompete the resistant bacteria once the drug is stopped.

## Cefiderocol selects for multiple resistance mutations

**Fig. 1 ∣:**
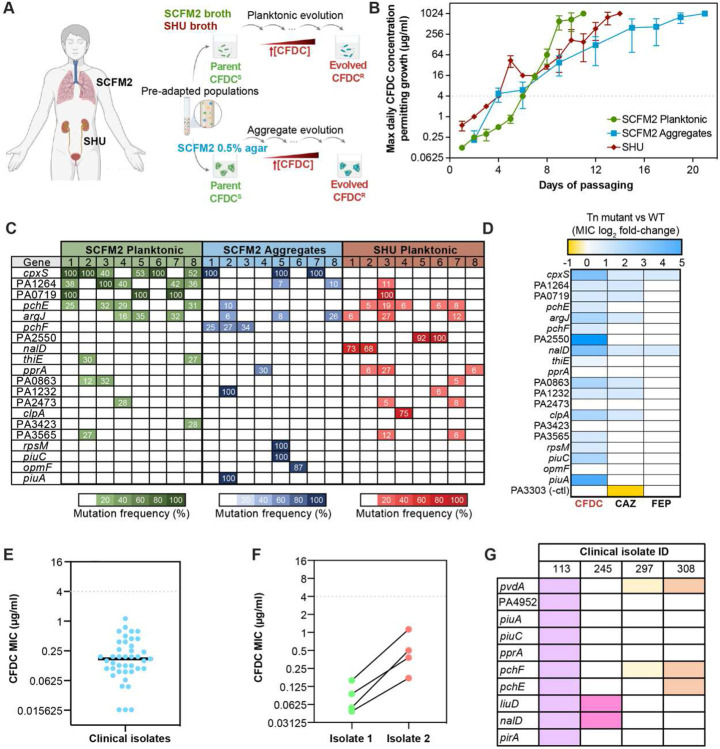
Cefiderocol resistance variants detected through experimental evolution are also detected in *P. aeruginosa* clinical isolates. **a**, Experimental design: *P. aeruginosa* PAO1 was propagated in SCFM2 or SHU for ten days prior to cefiderocol exposure. Pre-adapted populations were propagated daily in increasing cefiderocol concentrations planktonically in SHU or SCFM2 and as ABBA biofilm aggregates in SCFM2. When evolved population achieved growth at 1,024 μg/μl cefiderocol-evolved populations were harvested and sequenced to identify cefiderocol resistance mutations. As a control, 8 populations for each medium and lifestyle were propagated in antibiotic-free conditions. **b**, Cefiderocol resistance levels during experimental evolution. cefiderocol MICs were calculated using broth microdilution methods, (n=8, mean ± SEM). **c**, Heatmap showing mutations detected in populations which evolved to grow at 1,024 μg/μl cefiderocol in SCFM2 (planktonic and aggregates) and SHU. Variants detected in at least 25% of populations are listed. Each column represents one replicate population and color intensity indicates the maximum frequency of each gene variant detected in a given population. **d**, Heatmap showing antimicrobial susceptibilities of mutants carrying Tn insertions in candidate resistance genes identified via experimental evolution. Results indicate the average fold-change in MIC in the Tn-mutants compared to the PAO1 WT strain. cefiderocol, cefiderocol; CAZ, ceftazidime; FEP, cefepime. **e**, cefiderocol MICs of different clinical isolates recovered from patients with acute and chronic pneumonia that were never treated with cefiderocol (n=40). Each point represents the MIC from one clinical isolate. Dashed line indicates the MIC breakpoint for cefiderocol resistance. **f**, Increased cefiderocol MIC in paired sequential isolates. Even though no isolates were classified as cefiderocol resistant (MIC > 4 μg/ml) , we paired sequential clinical isolates recovered from the same patient which showed an increase in cefiderocol MIC. **g**, Heatmap indicating the presence of mutations in genes identified as being under selection during cefiderocol experimental evolution in clinical isolates with reduced cefiderocol susceptibility. Columns and colors represent each clinical isolate. Genomic DNA from clinical isolates were harvested, sequenced and chromosomal mutations were detected using Breseq.

We used experimental evolution to identify chromosomal mutations underlying cefiderocol resistance in *P. aeruginosa* in two different host mimicking media: synthetic cystic fibrosis sputum and synthetic human urine. We used planktonic conditions and biofilm aggregates to mimic modes of growth likely to occur in both infections (Extended Data Table 1). We pre-adapted the *P. aeruginosa* ancestor strain to each growth medium to reduce the influence of adaptation to the laboratory media on subsequent identification of resistance variants. Pre-adapted populations were passaged in increasing concentrations of cefiderocol either planktonically in broth or as aggregates in cystic fibrosis agar medium until each population achieved growth at 1,024 μg/ml cefiderocol (Fig. 1a). In parallel, bacteria were passaged without antibiotics as controls. Clinical resistance^7^ was achieved in less than one week in each medium: first in human urine, followed by cystic fibrosis aggregates and planktonic populations (Fig. 1b). The planktonic populations achieved high levels of cefiderocol resistance after 12-15 days of continuous passages (Fig. 1b), while cystic fibrosis aggregate populations achieved growth at 1,024 μg/ml more slowly after 20 days (Supplementary Table 5). These data showed that the nutritional environment and mode of growth affected rates of cefiderocol resistance evolution.

**Extended Data Figure 1 ∣:**
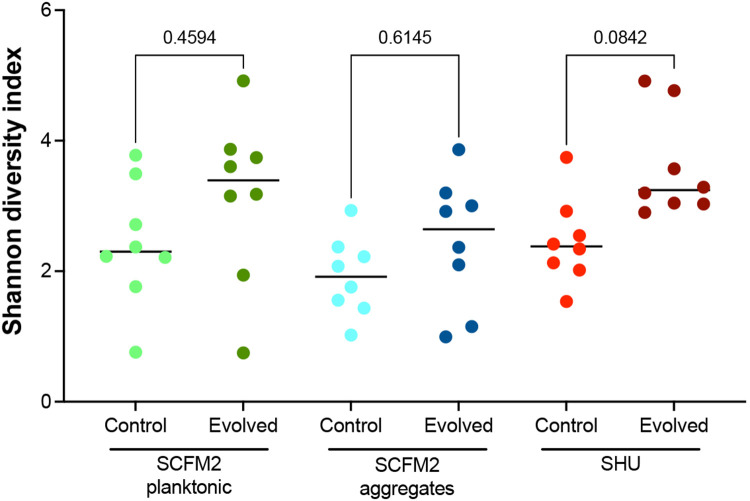
Genetic diversity of populations evolved in the presence or absence of increasing concentrations of CFDC. Shannon diversity indices were calculated from SNV frequencies in control and cefiderocol-evolved populations (mean; p-values, unpaired t-tests).

To determine cefiderocol resistance mutations, we performed whole genome sequencing on the evolved populations and compared them to the pre-adapted ancestral populations. Evolved populations were genetically diverse, exhibiting several mutations at intermediate frequencies, and fixed mutations were rare (Fig. 1c, Supplementary Table 1). Shannon diversity indices of evolved and control populations were not significantly different (Extended Data Fig. 1). Candidate cefiderocol resistance mutations affected some genes previously associated with cefiderocol resistance, including *piuA*^5^. Two genes were mutated in all host-mimicking conditions: *pchE* and *argJ*. Other mutations included those which were selected in distinct environments. Multiple mutations in the *cpxS* two component sensor gene were detected in cystic fibrosis-evolved populations, but no *cpxS* mutations were detected in urine-evolved populations. Mutations in PA0719, encoding a bacteriophage hypothetical protein, were fixed in some planktonic populations, but not in aggregates. Additionally, a large 21,938 bp deletion arose exclusively in populations growing in cystic fibrosis conditions. Together, these results suggest that some mutations contribute to cefiderocol resistance generally, while other mutations specifically increase resistance under certain nutrient or growth conditions.

Analyses of transposon mutants for genes that were mutated during experimental evolution showed that, in agreement with previous studies, *piuA* and *piuC* disruption resulted in a 4-fold increase of cefiderocol resistance^8^, while the PA3303 mutant, which served as a neutral Tn insertion negative control, did not exhibit increased cefiderocol resistance, or related antibiotics (Fig. 1d). As predicted, disruption of genes associated with iron acquisition, such as pyochelin (*pchEF*) siderophore genes led to 2- to 8-fold increases in resistance, as did mutations in *cpxS*, *nalD*, and PA2550. Unexpectedly, Tn insertions in *pprA*, PA3423, PA4952, and PA2473 had no effect on cefiderocol resistance. While it is unclear why these mutants did not increase cefiderocol resistance, it is possible that these represent compensatory mutations rather than direct cefiderocol resistance mutations. Together, these data suggest that high level cefiderocol resistance is achieved additively through multiple mutations, since no individual mutants displayed high resistance levels.

We next asked whether resistance mutations detected in the laboratory were also present in clinical isolates. We determined cefiderocol susceptibilities of 40 *P. aeruginosa* clinical isolates from 18 patients. While all the tested clinical isolates were clinically susceptible to cefiderocol (Fig. 1e)^7^, we identified four clonally related pairs of clinical isolates in which one isolate exhibited at least 3.5-fold higher cefiderocol MIC compared to the more susceptible isolate (Fig. 1f). Whole-genome sequencing showed that some of the recurrent mutations detected in the evolution experiment were also mutated in more resistant clinical isolates (Fig. 1g, Supplementary Data Table 2). Notably, these mutations primarily affected candidate resistance genes involved in iron acquisition, including iron-binding siderophore biosynthesis and receptor genes. These data suggest that host selective pressures affecting iron may also affect cefiderocol susceptibilities, even in the absence of cefiderocol treatment.

## Drug-free conditions restore susceptibility

**Fig. 2 ∣:**
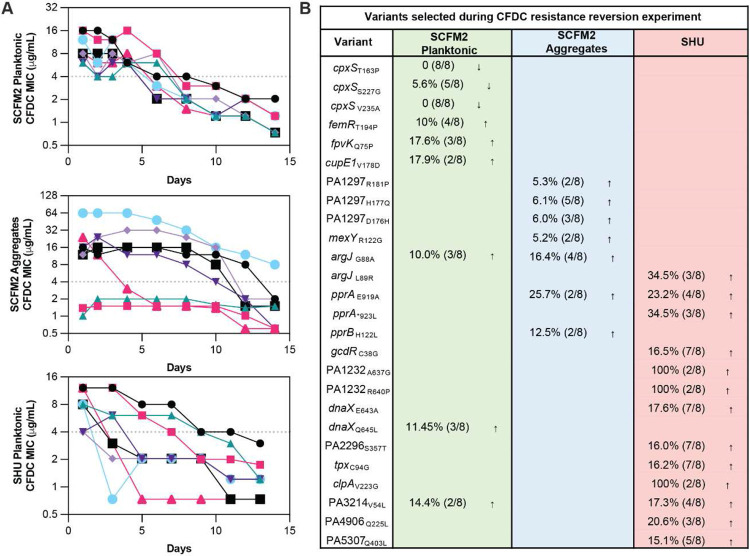
Cefiderocol resistant populations readily revert to cefiderocol susceptible in cefiderocol-free conditions. **a**, cefiderocol-resistant evolved populations (n=8) were continuously propagated for 14 days in cefiderocol-free SCFM2 or SHU. cefiderocol MICs were determined daily using cefiderocol gradient strips. The horizontal dashed line represents the cefiderocol susceptibility breakpoint (MIC < 4 μg/μl) defined by the FDA. **b**, Heatmaps indicate the genetic mutations the increased or decreased in prevalence after 15 days of propagating populations in cefiderocol-free media.

To test the durability of cefiderocol resistance evolution, we passaged cefiderocol-resistant populations in the absence of drugs and measured cefiderocol susceptibilities over time. All populations reverted to become cefiderocol susceptible, regardless of the medium or mode of growth (Fig. 2a), and susceptibility was restored fastest in aggregate populations, followed by cystic fibrosis and human urine planktonic populations (Supplementary Table 5). Whole-genome sequencing showed that reverted populations exhibited the 23-gene deletion that was recurrently observed during cystic fibrosis cefiderocol resistance evolution. This finding suggests that this deletion is an adaptation to the cystic fibrosis medium rather than a mutation associated with cefiderocol resistance. In the cefiderocol-free environment, *femR*, *fpvK*, *cupE1*, *argJ*, and *dnaX* were recurrently mutated in cystic fibrosis planktonic populations, whereas *PA1297*, *mexY*, *argJ*, *pprA*, and *pprB* mutations emerged in cystic fibrosis aggregates (Fig. 2b). Also, variants of *PA1232* and *clpA* were fixed across human urine populations propagated in the absence of cefiderocol. In contrast, *cpxS* variants were not maintained in cystic fibrosis planktonic populations evolved in the absence of cefiderocol, indicating that *cpxS* variants pay fitness costs in the drug-free environment (Fig. 2b). These findings motivated the investigation of the secondary phenotypic consequences of cefiderocol resistance.

## Cefiderocol selects for cross-resistance

To determine whether the evolution of cefiderocol resistance also affected susceptibility to other drugs, we measured susceptibilities of cefiderocol-evolved populations to a range of clinically relevant antibiotics. Compared with untreated control populations, cefiderocol-resistant evolved populations were more resistant to other cephalosporins, aztreonam, and tobramycin (Supplementary Table 5). In addition, cefiderocol-resistant populations evolved in cystic fibrosis conditions were more resistant to polymyxin B and colistin (Supplementary Table 5). Populations evolved in urine mimicking conditions were slightly more resistant to ciprofloxacin (Supplementary Table 5). Collateral susceptibility was not observed for any antibiotics tested.

## Cefiderocol resistance pays fitness costs

**Fig. 3 ∣:**
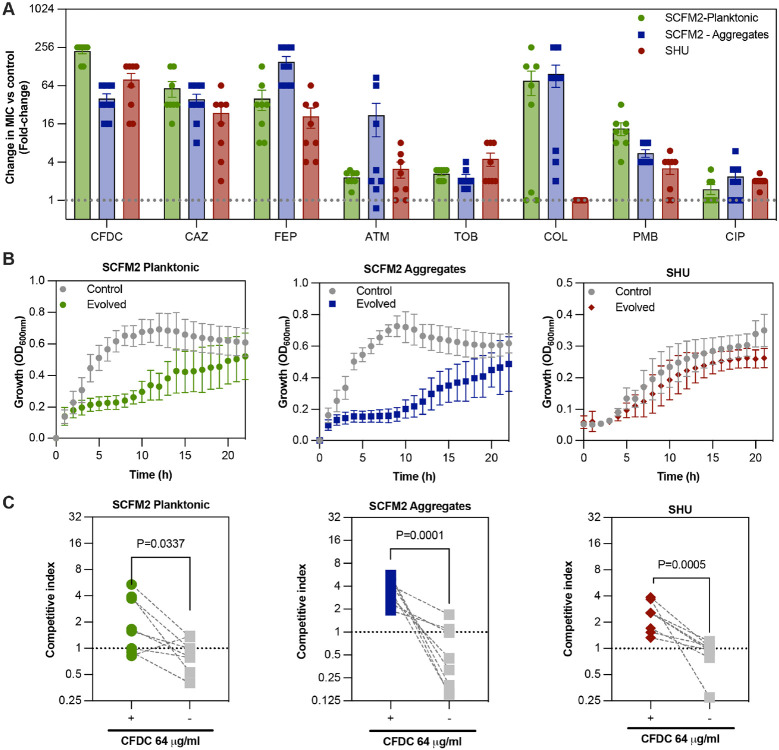
Fitness trade-offs evolved in cefiderocol-resistant populations. **a**, Antimicrobial susceptibility testing of cefiderocol resistant populations compared with untreated control populations. The resistance profiles were analyzed by broth microdilution assays (n=3). Results indicate mean fold-change in MIC comparing control and evolved populations. **b**, Growth curves of evolved and untreated control populations in the absence of cefiderocol. Evolved populations were grown in SCFM2 or SHU in the absence of cefiderocol for 24h and growth was measured every 30 min by absorbance at 600 nm (n=3). Doubling times: SCFM2 planktonic evolved 268.42 ± 132.91 m vs control 56.16 ± 13.27 m, p=0.0129; SCFM2 aggregate evolved 394.80 ± 215.8 m vs control 47.39 ± 7.59 m, p=0.0026; paired t-test. doubling times: SHU evolved: 173.83 ± 89.74 m vs SHU control: 91.79 ± 30.16 m, p=0.2827; paired t-test. **c**, *In vitro* competition between ancestral and evolved populations. Evolved and ancestral populations were tagged with eYFP and mApple fluorescent proteins, respectively. The fluorescent populations were competed (at 1:1 ratio) in the presence or absence of cefiderocol. CI > 1 indicates that evolved populations outcompeted their ancestors. Experiments were performed in triplicate, in three independent experimental sets (mean ± SD). cefiderocol, cefiderocol; CAZ, ceftazidime; FEP, cefepime; ATM, aztreonam; TOB, tobramycin; COL, colistin; PMB, polymyxin B; CIP, ciprofloxacin.

Considering that cefiderocol resistance was lost in the absence of antibiotic pressure, we hypothesized that cefiderocol resistance would pay fitness costs in the absence of cefiderocol. Indeed, highly resistant populations selected in cystic fibrosis conditions exhibited significantly slower growth rates (Fig. 3b and Supplementary Table 5). In contrast, evolved populations selected in urine medium did not exhibit significant growth defects (Supplementary Table 5).

To further investigate the relationship between cefiderocol resistance and fitness, we tested the competitiveness of evolved populations against their ancestors in the presence or absence of cefiderocol. In the absence of cefiderocol, ancestral populations outcompeted the cefiderocol-resistant evolved populations in the cystic fibrosis environment. However, in urine co-culture both evolved and ancestral populations grew at equal rates, indicating that cefiderocol-resistant mutations acquired in urine medium paid little or no fitness cost in the absence of cefiderocol (Fig. 3c and Extended Data Fig. 3).

## Cefiderocol selects for cross-protection

**Extended Data Figure 2 ∣:**
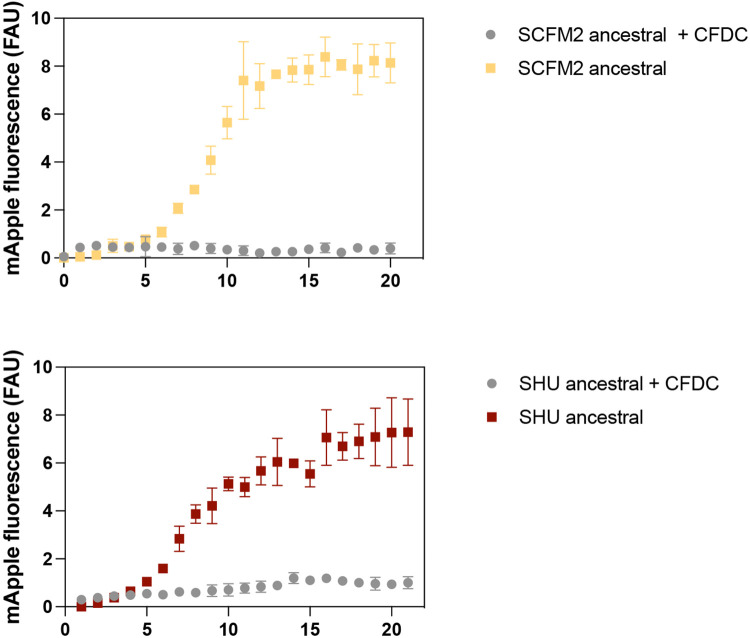
Ancestral populations are not able to grow in presence of cefiderocol. Growth in the presence and absence of 64 μg/ml cefiderocol of populations was determined by monitoring fluorescence over time (h).

**Extended Data Figure 3 ∣:**
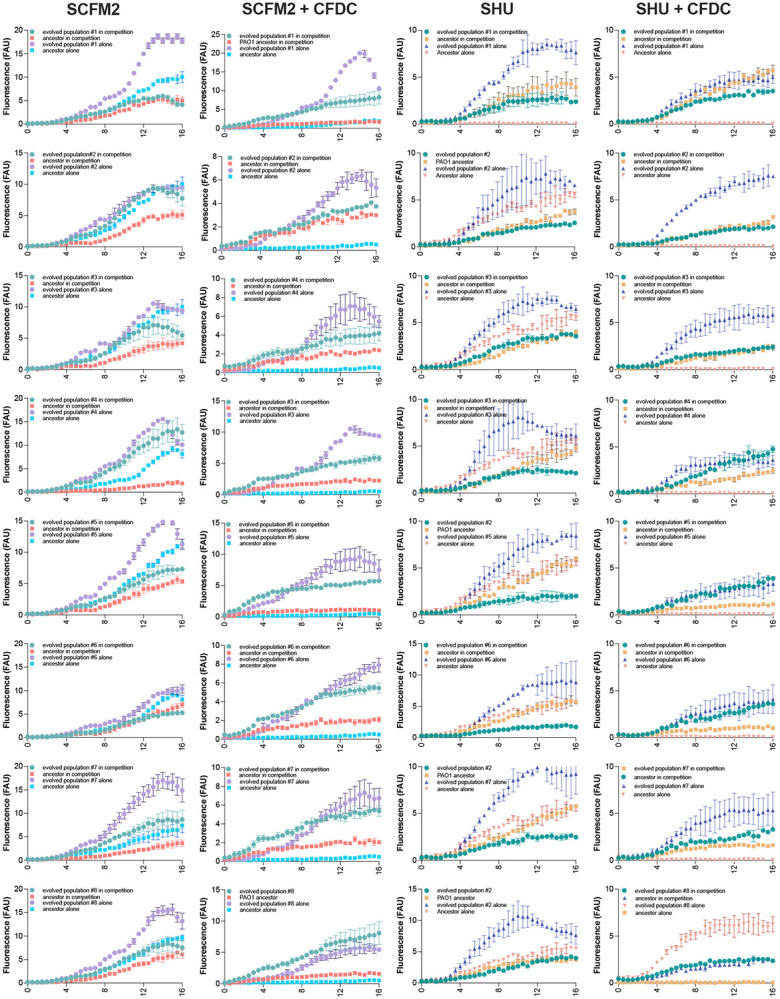
*In vitro* competition between ancestral and CFDC-resistant evolved planktonic populations. Evolved and ancestral populations were tagged with eYFP and mApple fluorescent proteins, respectively. The fluorescent populations were competed (at 1:1 ratio) in the presence or absence of cefiderocol (64 μg/ml). Populations growth was determined by monitoring fluorescence over time (h). Experime were performed in triplicate, in three independent experimental sets (mean ± SD).

**Extended Data Figure 4 ∣:**
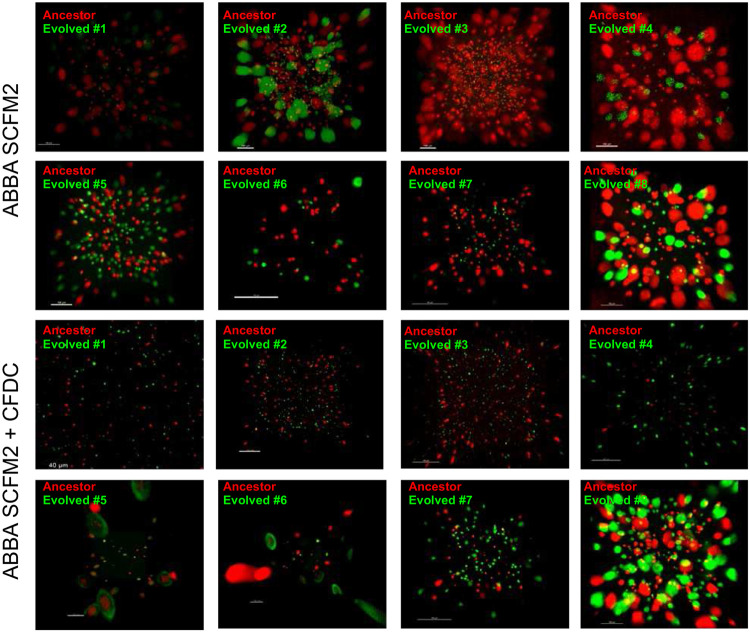
*In vitro* competition between ancestral and CFDC-resistant evolved aggregates populations. Evolved and ancestral populations were tagged with eYFP and mApple fluorescent proteins, respectively. The fluorescent populations were competed (at 1:1 ratio) in the presence or absence of cefiderocol (64 μg/ml). Aggregate populations were visualized with a Zeiss LSM 780 confocal microscope.

**Fig. 4 ∣:**
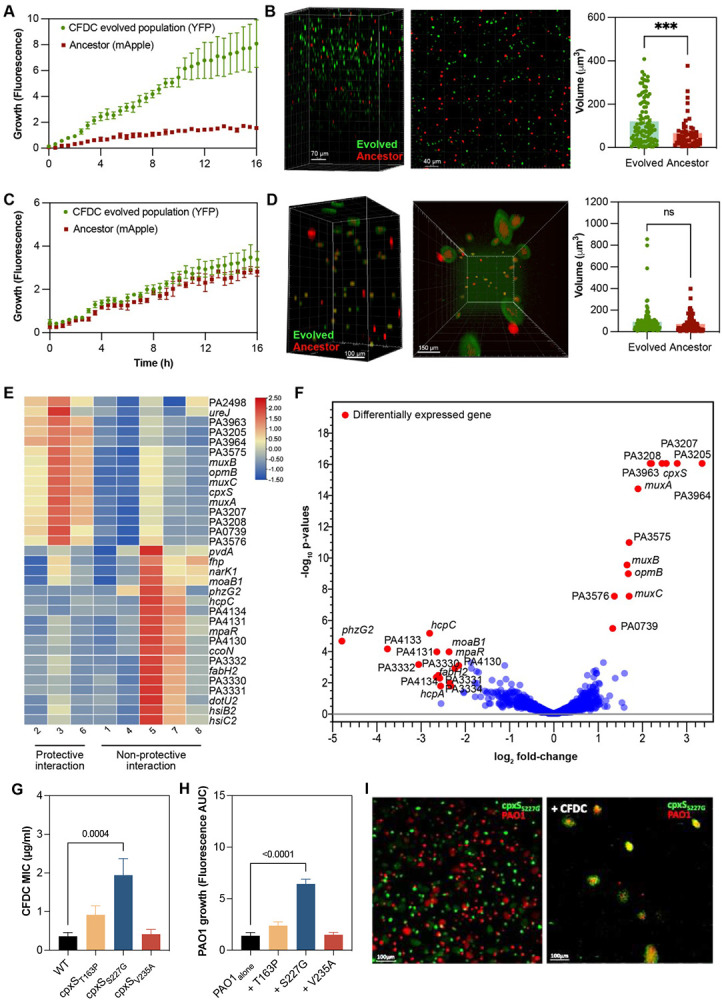
Cross-protection evolved in cefiderocol-resistant populations. **a-d**, As expected, the *in vitro* competition assay revealed that most evolved populations outcompeted ancestral populations during competition in SCFM2 under high cefiderocol pressure (64 μg/ml) both in planktonic (**a**) and in biofilm conditions (**b**). However, some cefiderocol-resistant evolved populations evolved benevolent social behavior allowing the ancestral cefiderocol-susceptible siblings to survive cefiderocol insult under well-mixed (**c**) and structured environments (**d**). Figures (**b**) and (**b**) show representative 3D confocal micrographs of non-protective and protective interactions between cefiderocol-resistant evolved populations (green – eYFP) and ancestral susceptible populations (red – mApple) in the presence of cefiderocol (64 μg/μl). To understand which traits drive the protective benevolent behavior, we compared the differential expression profiles associated with benevolent behavior observed during protective and non-protective competition under cefiderocol pressure (64 μg/ml). **e**, Heatmap showing differential expression in protective and non-protective populations. The gradient from blue to red indicates the relative degree of expression of DEGs (>1.5 log_2_ fold-change, FDR < 0.05). The outer circle labels represent the gene name or locus tag for each DEG. **f**, Volcano plot highlighting the differentially expressed genes identified using CLC genomics by comparing the average of protective and non-protective competitions. The red dots represent the DEGs (adjusted P-value < 0.05 with log_2_ fold-change > 1.5). **g**, cefiderocol MIC of PAO1 and *cpxS* variants (mean ± SEM, ANOVA). **h**, Planktonic competitions of PAO1 and *cpxS* variants (T163P, S227G, V235A) in SCFM2 with ½ × MIC. The growth of PAO1 was measured by mApple fluorescence area under the curve (AUC). **i**, Biofilm competition between PAO1 and *cpxS_S227G_* in SCFM2 in the absence and presence of 2 μg/ml cefiderocol.

Each cefiderocol-evolved populations’ fitness was also evaluated in the presence of cefiderocol. As expected, all urine-adapted cefiderocol-resistant populations were more fit in high cefiderocol concentrations (64 μg/ml) (Fig. 3c and Extended Data Fig. 3). Moreover, 5 out of 8 CF-evolved populations outcompeted the susceptible populations when cefiderocol was present (Fig. 3c and Fig. 4a-b). Unexpectedly, three evolved cystic fibrosis planktonic populations grew at the same rate as ancestral susceptible populations during cefiderocol co-culture (Fig 4c and Extended Data Fig. 3). The growth of the ancestral susceptible populations was surprising because it cannot grow in high cefiderocol concentrations in isolation (Extended Data Fig. 2). The unexpected growth during cefiderocol exposure indicated that “benevolent” cross-protective subpopulations produced a public good which allowed cefiderocol-susceptible siblings to grow in otherwise lethal cefiderocol concentrations. The protective phenotype was also observed in the aggregate conditions, where cefiderocol-resistant evolved populations grew in the outermost layers of the aggregate, theoretically shielding the cefiderocol-susceptible ancestral population from cefiderocol killing (Fig. 4d and Extended Data Fig. 4). Such benevolent behaviors did not arise in populations evolved under urine conditions, indicating this social trait is favored in a cystic fibrosis environment.

## Sensor variants drive cross-protection

We next asked which *P. aeruginosa* traits drove the cross-protection of cefiderocol-susceptible siblings. We compared the gene expression profiles of protective versus non-protective co-cultures in the presence of cefiderocol using RNA-seq. These transcriptome analyses revealed that protective co-cultures had a unique gene expression pattern: 34 genes were differentially expressed, including 16 upregulated genes and 18 downregulated genes (Fig. 4e-f, Supplementary Table 4). Surprisingly, no classical public goods related to β-lactam degradation, such as β-lactamases, were increased in expression in the protective co-cultures. However, protective co-cultures upregulated the expression of the CPX two-component system genes and the *muxABC-opmB* efflux pump. MuxABC-OpmB plays a role in resistance to novobiocin, aztreonam, macrolides, and tetracycline, and its overexpression is associated with modest increases in pyoverdine secretion^9,10^.

The upregulation of genes under control of the CPX two-component system motivated us to further analyze its role in benevolent cross-protection. We tested the ability of three *cpxS* variants to cross-protect the parental strain. While the *cpxS_T163P_* and *cpxS_V235A_* variations had, respectively, little effect on cefiderocol resistance, the *cpxS_S227G_* variant induced a 5-fold increase in cefiderocol MIC (Fig. 4g). Moreover, the *cpxS_S227G_* was able to crossprotect PAO1 from cefiderocol killing (Fig. 4h-i). Cross-protective behavior in biofilm communities has been primarily associated with β-lactamase mediated drug inactivation^1,2^. However, *ampC* was not differentially expressed during cross-protective interactions. Considering the upregulation of MuxABC-OpmB (Fig. 4e-f) during cross-protective interactions and its role in pyoverdine secretion, we reasoned that pyoverdine secreted by MuxABC-OpmB could act as a public good protecting the susceptible siblings.

**Extended Data Figure 5 ∣:**
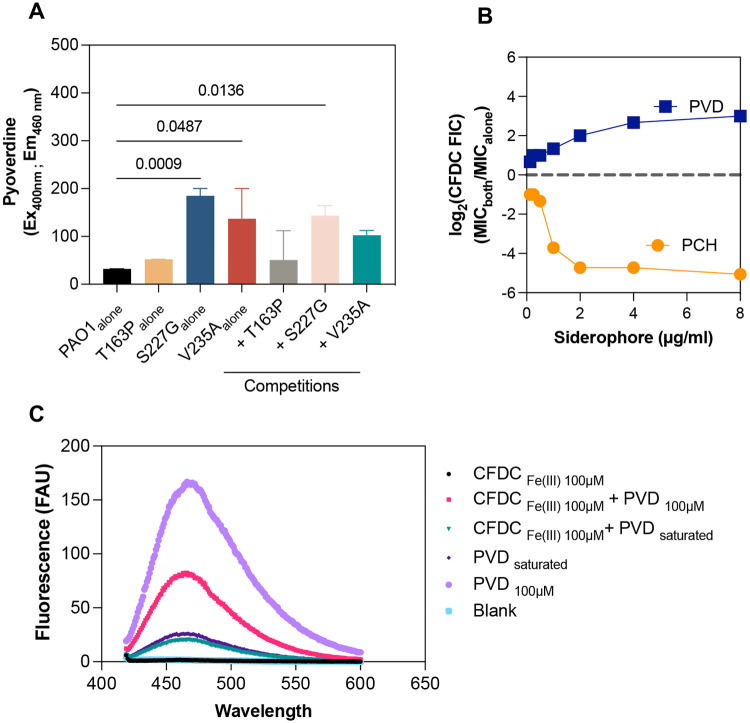
The role of pyoverdine in benevolent cross-protection. **a**, Pyoverdine production by PAO1 and *cpxS* variants (T163P, S227G and V235A) in SCFM2 with ½ × CFDC MIC measured after 20 h of incubation (mean ± SEM, ANOVA). **b**, Changes in CFDC susceptibility in the presence of pyoverdine (PVD) and pyochelin (PCH), the combinatorial effect is expressed by the log_2_ CFDC fractional inhibitory concentration (FIC). **c**, To measure the displacement of Fe(III) from CFDC, 100 μM of CFDC was pre-incubated with 100 μM of FeCl_3_ for 16h at room temperature. Next, 100 μM of free pyoverdine was added to ferric-CFDC and incubated for an additional hour. Iron(III) displacement was determined by pyoverdine fluorescence quenching.

In the presence of 1/2 MIC of cefiderocol *cpxS_S227G_* (alone or in co-culture with PAO1) increased pyoverdine production compared to PAO1 growing alone (Extended Data Fig. 5a). To confirm the effect of pyoverdine on cefiderocol efficacy, we performed checkerboard assays combining cefiderocol and *P. aeruginosa* siderophores (pyoverdine and pyochelin). The siderophores displayed opposite effects on cefiderocol activity. While cefiderocol and pyochelin were synergistic, pyoverdine antagonistically decreased cefiderocol activity against PAO1 (Extended Data Fig. 5b). Previously, it was shown that pyoverdine had a greater ability to chelate Fe(III) than cefiderocol^3^. Hence, we hypothesized that pyoverdine attenuates cefiderocol activity by displacing Fe(III) from the cefiderocol catechol group. Iron displacement was measured by fluorescence quenching of pyoverdine, and at equimolar amounts pyoverdine was able to chelate Fe(III) from ferric-cefiderocol (Extended Data Fig. 5c). Together these results support the hypothesis that cefiderocol cross-protection is conferred by increased pyoverdine secretion of *cpxS* variants.

## Discussion

Using experimental evolution, we investigated the molecular mechanisms of cefiderocol resistance and the secondary phenotypic effects of cefiderocol resistance in *P. aeruginosa*. Populations in cystic fibrosis- and urine-mimicking media achieved high levels of cefiderocol resistance; however, both the nutritional environment and the mode of growth (e.g., planktonic vs. aggregate growth) affected the rates of cefiderocol resistance evolution. In contrast to evolution in rich liquid growth media where typically a few strains are selected for and dominate each population^11,12^, cefiderocol-evolved populations from host-mimicking media were highly diverse with few mutations that swept the populations. Several genes were recurrently mutated in multiple populations and this parallelism reflects natural selection^13^. These mutations included genes previously implicated in cefiderocol resistance (e.g., *piuA*), as well as genes not linked to cefiderocol resistance (e.g., *cpxS*). A secondary consequence of cefiderocol resistance evolution was the development of multidrug resistance, which raises concerns about cefiderocol use in patient care. However, these concerns are somewhat tempered by 1) fitness costs paid by cefiderocol-resistant strains that are outcompeted by susceptible siblings in the absence of cefiderocol and 2) experiments showing that cefiderocol susceptibility could be restored in populations passaged in cefiderocol-free conditions. Concerns remain however, because some resistant populations cross-protected susceptible siblings during cefiderocol exposure.

Some populations in cystic fibrosis conditions evolved benevolent cross-protective cooperative behavior. Cross-protection mediated by β-lactamases has been described^1,2^; however, our findings indicate that cefiderocol cross-protection is AmpC-independent. In protective co-cultures, *cpxS* and the *cpxR*-regulated *muxABC-opmB* efflux pump ^14^ were overexpressed. We showed that the *cpxS_S227G_* variant cross-protected WT PAO1, suggesting that conformational changes of the CpxS sensor kinase might lead to CPX hyperactivation. Previously, mutations of the *cpxS* homolog *cpxA* were shown to prevent its interaction with CpxP, resulting in continuous CPX activation^15^. We hypothesized that increased *muxABC-opmB* expression could drive benevolent cross-protection of susceptible siblings by increasing pyoverdine production. While not required for siderophore secretion, MuxABC-OpmB contributes to the secretion of coumarin-containing compounds, including pyoverdine^10^. In line with this, we found that *cpxS* variants secreted more pyoverdine and we showed that pyoverdine could chelate iron from cefiderocol. An alternate possibility is that MuxABC-OpmB acts directly on cefiderocol, pumping cefiderocol out of aggregate structures and reducing drug concentrations in the internal core of the aggregates. In support of this second model, public good producing cells in aggregates proliferate faster under antibiotic pressure and localize on the edge of biofilm colonies^2,17,18^. One clear limitation of this experiment is that we were not able to completely sort evolved and ancestor populations. Because of that, it was not possible to distinguish whether differentially expressed genes were coming from resistant or susceptible cells within the populations. Therefore, future work will need spatially-resolved single-cell expression analyses to identify molecular mechanisms of benevolent cross-protection in cefiderocol-resistant populations.

## Methods

### Strains, media, and antibiotics

*P. aeruginosa* strain PAO1 was used for all cefiderocol resistance evolution experiments. SCFM2 was prepared as previously described ^19^. Synthetic human urine was prepared as previously described but without adding FeSO_4_^20^. Bacterial populations were grown at 37°C in SCFM2 or SHU media as specified. cefiderocol was obtained from MedChem Express (Monmouth Junction, NJ, USA). PAO1 transposon mutants were acquired from the Manoil laboratory (University of Washington) ^21^. The identity of all PAO1 transposon mutants was confirmed using insertion-specific PCR before use.

### Experimental evolution

PAO1 was propagated for ten days in SCFM2 and SHU conditions in preparation for the evolutionary experiment. The overnight cultures were diluted (1:100) into fresh medium without cefiderocol and grew overnight. This passaging step aimed to preadapt the lab strain PAO1 to the CF- or UTI-like states and reduce the influence of adaptation to the host mimicking media in the following comparisons ^22^.

After 10 days of preadaptation, SCMF2- or SHU-adapted populations were subcultured into eight replicate populations that were propagated daily with increasing concentrations of cefiderocol. Each day, a 2-fold serial dilution of cefiderocol was freshly prepared in SCFM2 or SHU with concentrations ranging from 0.003-1024 mg/ml cefiderocol, and 5 μl of aerobically grown overnight planktonic cultures were inoculated into each well. After overnight aerobic incubation in a cefiderocol-containing medium, cells from the highest concentration of cefiderocol which supported growth were harvested. As before, 5 μl of this culture was reinoculated into aliquots of fresh cefiderocol-containing medium, and the remaining cells were frozen with 25% of glycerol at −80°C.

SCFM2 preadapted PAO1 was used to evolve cefiderocol resistance in SCFM2 aggregate populations. To mimic the oxygen and nutrient-limiting gradients that structured bacterial populations face during chronic CF infections, we used the agar biofilm block assay (ABBA) ^23^. SCFM2-preadapted populations were grown overnight at 37°C and 250 RPM. Cultures were diluted to OD_600_ of 0.002 in molten SCFM2 with 0.5% noble agar, and 100 μl of the bacterial agar suspension was transferred to 96-well plate containing 100 μl 2-fold serial dilutions spanning ten cefiderocol dilutions in SCFM2, including concentrations ranging from 0.003-1024 mg/ml cefiderocol. After agar solidified at 25°C, bacterial aggregates were transferred to a humidified chamber and incubated statically at 37°C. After overnight growth, aggregates from the highest concentration of cefiderocol which supported growth were washed twice with 1× PBS to wash away cells growing on the agar block surface. Then, aggregates were mechanically disrupted by vigorous pipetting with 100 μl PBS. Disrupted aggregates were centrifuged at 5000 × g for 10 m and resuspended in 500 μl SCFM2. As described above, aggregated cells were diluted into molten SCFM2 agar and reinoculated into a new cefiderocol-containing SCFM2 agar plate. Remaining cells were frozen with 25% of glycerol.

Planktonic and biofilm populations were propagated with increasing drug concentrations until achieving growth at 1,024 μg/ml cefiderocol. In parallel, 8 replicates of preadapted populations were passaged planktonically in SCFM2 and SHU and as aggregates in SCFM2 without any antibiotic as a control.

### Genome sequencing and analysis

DNA from control and evolved populations was extracted using the Qiagen DNeasy Blood and Tissue kit (Qiagen, Hiden, Germany). Sequencing libraries were prepared using the Illumina DNA Prep kit (Illumina Inc., San Diego, CA) and sequenced using an Illumina NovaSeq6000 at the Cedars-Sinai Cancer Applied Genomics Core. The variants were called using Breseq software package v0.31.0 using the default parameters and the -p flag for estimating the polymorphisms frequencies in populations using the *P. aeruginosa* PAO1 genome as a reference ^24^ After the variant calling, the variants detected in both control populations and in cefiderocol-evolved populations were manually removed from further consideration. Mutations detected in at least 25% of any replicate were selected and summarized in Fig 1c, and all mutations detected in cefiderocol-evolved populations, but not in control populations, are shown in Supplementary Table 1. The genetic diversity across evolved populations was estimated by the calculation of Shannon index considering the presence and frequency of mutated genes. Shannon indices were calculated using vegan 2.6-4 R package^25^. *P. aeruginosa* clinical isolates (n=40) were kindly provided by the Pulmonary Translational Research Core at University of Pittsburgh. These isolates were sequenced by the Microbial Genome Sequencing Center (MiGs) and indels and polymorphisms were called using Breseq as described above but using the consensus mode.

### Cefiderocol susceptibility testing of *P. aeruginosa* clinical isolates

cefiderocol resistance levels of *P. aeruginosa* clinical isolates (n=40) were determined using cefiderocol MIC test strips (Liofilchem) placed onto cation-adjusted Muller-Hinton agar following the manufacturer’s instructions. Isolates’ cefiderocol susceptibility levels were classified following the FDA breakpoints (sensitivity ≤1 μg/ml, Intermediate 2 μg/ml, resistance ≥ 4 μg/ml) ^7^. Of note, current CLSI breakpoint for cefiderocol classifies *P. aeruginosa* as susceptible, intermediate, and resistant isolates with MIC ≤4, 8 and ≥16 μg/ml, respectively ^26^ Pairs of clinical isolates obtained from the same patient which presented with at least a 3.5-fold increase in cefiderocol MIC had their genomes analyzed as described above, and mutations detected in paired clinical isolates analyzed are shown in Supplementary Table 2.

### Antibiotic susceptibility testing

Antibiotic susceptibility profiles of the control and evolved populations were determined by broth microdilution assay according to the Clinical and Laboratory Standards Institute guidelines, in which each bacterial isolate was tested in 2-fold-increasing concentrations of cefiderocol, CAZ, FEP, ATM, TOB, COL, PMB and CIP. Cells were incubated at 37°C for 24 h and the lowest concentration of each antibiotic that inhibited bacterial growth, as determined by the absence of visible turbidity in each well, was considered the minimal inhibitory concentration (MIC). Antibiotic susceptibility testing was performed using cation-adjusted Mueller-Hinton broth (CAMHB), except for cefiderocol MIC assays where iron-depleted CAMHB was used. Iron-depleted CAMHB was prepared by adding 100 g of chelex-100 resin per liter of autoclaved CAMHB and the suspension was stirred for 2 h at room temperature to remove cations in the medium. The depleted broth was filtered to remove the chelex resin and supplemented with 22.5 μg/ml CaCl_2_, 11.25 μg/ml MgCl_2_ and 56 μg/ml ZnSO_4_ (pH 7.2). Finally, the iron-depleted CAMHB was filter-sterilized and stored at room temperature.

### *In vitro* competition assays

To compare relative fitness of cefiderocol resistance mutations, we performed *in vitro* competitions between ancestral and evolved final populations, tagged with mApple and eYFP fluorescent proteins, respectively. For competitions, each planktonic population was propagated from a freezer stock in appropriate conditions (media and cefiderocol concentration) for 24 h. Both ancestor and evolved populations were tagged by integrating mApple (red fluorescent protein) or eYFP (yellow fluorescent protein) at the *att* locus using the broad host-range mini-Tn7 vectors following the protocol described elsewhere ^27^. Briefly, 1.5 mL of ancestral and cefiderocol-evolved populations grown overnight appropriate conditions were centrifuged at 14,000× *g* for 3 m and washed twice with 300 mM sterile sucrose. Ancestral or cefiderocol-evolved populations were electroporated with 500 ng of pDB809 PrpsG::mApple or pUC18T-miniTn7T-Gm-PA1/04/03/eyfpT0T1, respectively, in combination with 500 ng of helper plasmid pTNS2. Transformants grown in the presence of 30 μg/ml gentamicin were pooled together for reconstituting the mixed populations. Overnight fluorescent cultures were diluted to OD_600_ ~0.005 (~5 × 10^6^ CFU/mL) and mixed in a 1:1 ratio. After mixing ancestor and evolved populations, we inoculated 10 μl of mixed or single bacterial suspensions in 100 μl SCFM2 or SHU with or without 64 μg/ml cefiderocol in triplicate in a sterile 96-well plate. 50 μl of mineral oil was added to each well to prevent evaporation. The plates were incubated for 20 h at 37°C, shaking at 250 rpm, and the eYFP (excitation: 510 ± 5 nm/ emission: 534 ± 5 nm) and mApple (excitation: 568 ± 5 nm, emission: 592 ± 5 nm) fluorescence was monitored hourly for 20 h using a Varioskan Lux Microplate Reader (Thermo Fisher Scientific, Waltham, Massachusetts). Planktonic competitive indices (CI) were calculated following the equation 1:

(1) PlanktonicCI=∕AUCeYFPaloneAUCeYFPcompetition∕AUCmApplealoneAUCmApplecompetition

For aggregate competition assays, cefiderocol-evolved aggregate populations were tagged with eYFP as described above. Overnight fluorescent cultures were diluted to OD_600_ ~0.1 and mixed at 1:1 ratio. Then, 10 μl of mixed and single cultures were added and mixed into 1 ml SCFM2 with 0.5% noble agar for a final starting dilution of OD_600_ of 0.001. 200 μl of the bacterial agar suspension was transferred into a well of an eight-well glass chamber slide for microscopy (Thermo Fisher Scientific, Waltham, Massachusetts). After overnight growth, aggregates from the highest concentration of cefiderocol which supported growth were washed twice with 1× PBS to remove planktonic cells growing on the agar block surface. Aggregate populations were visualized with a Zeiss LSM 780 confocal microscope in the Cedars-Sinai Microscopy Core Facility. 500-μm z-stacks (50 slices total, 10-μm step size) were collected in 8-bit mode with a scan format of 512 × 512 pixels ^23^. Aggregate volume was quantified using Imaris 9.9 (Oxford Instruments) using the surfaces function in Imaris based on the mApple or eYFP signals in each Z-stack as previously described ^28^. Aggregate CI was calculated following the equation 2:

(2) AggregateCI=∕volumeeYFPalonevolumeeYFPcompetition∕volumemApplealonevolumemApplecompetition

### RNA sequencing analyses

RNA sequencing analysis was performed to measure global gene expression in the ancestor and evolved planktonic populations grown in co-culture. Ancestral populations were co-cultured in competition with each evolved population in SCFM2 with 64 μg/ml cefiderocol as described above. After 8 h of competition, cells were harvested and the RNA was extracted using a RNeasy mini Kit (Qiagen, Hiden, Germany). Residual DNA was removed using a DNA-free DNA Removal Kit (Invitrogen, Waltham, Massachusetts). Ribosomal RNA was depleted with the Ribo-Zero rRNA Removal Kit (Illumina). RNA sequencing libraries were prepared using the Illumina Stranded Total RNA Prep kit (Illumina Inc., San Diego, CA) and sequenced using an Illumina NovaSeq6000 at the Cedars-Sinai Cancer Applied Genomics Core targeting 30M reads per sample.

RNA-seq reads were analyzed using CLC Genomics workbench 22.01 (Qiagen). The *P. aeruginosa* PAO1 genome and annotations were downloaded from the Pseudomonas Genome Database and used as a reference for analyses. RPKM values were generated using default parameters. Fold changes in gene expression and statistical analyses were performed using an Extraction of Differential Gene Expression (EDGE) test with the Bonferroni correction.

## Extended Data

**Extended Data Table 1.** SCFM2 and SHU chemical composition

SCFM2 chemical composition:		SHU chemical composition:	
Chemical	Final concentration	Chemical	Final concentration
NaH2PO4	1.3 mM	NaCl	100 mM
Na2HPO4	1.25 mM	Na_2_SO_4_	17 mM
KNO3	0.348 mM	Urea	280 mM
K2SO4	0.271 mM	KCl	38 mM
NH4C1	2.280 mM	CaCl_2_	4 mM
KCl	14.942 mM	Creatinine	9 mM
NaCl	51.848 mM	Na_3_C_6_H_5_O_7_	3.4 mM
MOPS	10 mM	NH_4_Cl	20 mM
Ser	1.446 mM	MgSO_4_	3.2 mM
Glu * HCl	1.549 mM	Na_2_C_2_O_4_	0.18 mM
Pro	1.661 mM	NaH_2_PO_4_	3.6 mM
Gly	1.203 mM	Na_2_HPO_4_	6.5 mM
Ala	1.78 mM	KH_2_PO_4_	16 mM
Val	1.117 mM	C_5_H_4_N_4_O_3_	0.6 mM
Met	0.633 mM	NaHCO_3_	13.5 mM
Ile	1.121 mM	MgCl_2_.6H_2_O	3.2 mM
Leu	1.609 mM	C_3_H_6_O_3_	1.1 mM
Orn * HCl	0.676 mM		
Lys * HCl	2.128 mM		
Arg * HCl	0.306 mM		
Trp	0.013 mM		
Asp	0.827 mM		
Tyr	0.802 mM		
Thr	1.072 mM		
Cys * HCl	0.16 mM		
Phe	0.53 mM		
His * HCl * H2O	0.519 mM		
Salmon Sperm DNA	0.6 mg/mL		
Bovine Maxillary Mucin	5 mg/mL		
Dextrose (D-glucose)	3 mM		
L-lactic acid	9.3 mM		
CaCl2 * 2H2O	1.754 MM		
MgCl2 * 6H2O	0.606 mM		
FeSO4 * 7H2O	0.0036 mM		
N-acetylglucosamine	0.3 mM		
1,2-dioleoyl-sn-glycero-3-phosphocholine (DOPC)	100 μg/mL		

## Supplementary Material

1
