# Supplementary material for: Evolved bacterial siderophore-mediated antibiotic cross-protection
Source: Res Sq. 2023 May 18:rs.3.rs-2644953. Preprint. [Version 1] doi: 10.21203/rs.3.rs-2644953/v1 (PMC10246284; doi:10.21203/rs.3.rs-2644953/v1)
Supplement: 1 [file NIHPPrs2644953v1-supplement-1.pdf]

Supplementary Information Guide

Supplementary Table 1: Complete list of mutated genes in the evolved population with increasing concentrations of CFDC. Mutations detected in ancestral and control populations were filtered.

Supplementary Table 2: Complete list of mutated genes in clinical isolates sequentially recovered from ICU-admitted patients infected with *P. aeruginosa.*

Supplementary Table 3: Complete list of mutated genes evolving in CFDC-resistant population propagated for 14 days in absence of CFDC.

Supplementary Table 4: Differentially expressed genes in protective interactions compared with non-protective interactions.

Supplementary Table 5: Summary statistics analysis

Supplementary Files

This is a list of supplementary files associated with this preprint. Click to download.

- SupplementaryTable1.xlsx
- SupplementaryTable2.xlsx
- SupplementaryTable3.xlsx
- SupplementaryTable4.xlsx
- SupplementaryTable5.xlsx
